# Comparative Genomic In Situ Hybridization and the Possible Role of Retroelements in the Karyotypic Evolution of Three Akodontini Species

**DOI:** 10.1155/2017/5935380

**Published:** 2017-08-15

**Authors:** Naiara Pereira Araújo, Gustavo Campos Silva Kuhn, Flávia Nunes Vieira, Thaís Queiroz Morcatty, Adriano Pereira Paglia, Marta Svartman

**Affiliations:** ^1^Laboratório de Citogenômica Evolutiva, Departamento de Biologia Geral, Instituto de Ciências Biológicas, Universidade Federal de Minas Gerais, Avenida Presidente Antônio Carlos, 6627-Pampulha, 31270-901 Belo Horizonte, MG, Brazil; ^2^Laboratório de Ecologia e Conservação, Departamento de Biologia Geral, Instituto de Ciências Biológicas, Universidade Federal de Minas Gerais, Avenida Presidente Antônio Carlos, 6627-Pampulha, 31270-901 Belo Horizonte, MG, Brazil

## Abstract

South American Akodontini rodents are characterized by a large number of chromosome rearrangements. Among them, the genus *Akodon* has been extensively analyzed with classical and molecular cytogenetics, which allowed the identification of a large number of intra- and interspecific chromosomal variation due to Robertsonian rearrangements, pericentric inversions, and heterochromatin additions/deletions. In order to shed some light on the cause of these rearrangements, we comparatively analyzed the karyotypes of three Akodontini species, *Akodon cursor* (2n = 14, FN = 19), *A. montensis* (2n = 24, FN = 42), and *Necromys lasiurus* (2n = 34, FN = 34), after GTG- and CBG-banding. The karyotypes differed by Robertsonian rearrangements, pericentric inversions, centromere repositioning, and heterochromatin variation. Genome comparisons were performed through interspecific fluorescent in situ hybridization (FISH) with total genomic DNAs of each species as probes (GISH). Our results revealed considerable conservation of the euchromatic portions among the three karyotypes suggesting that they mostly differ in their heterochromatic regions. FISH was also performed to assess the distribution of telomeric sequences, long and short interspersed repetitive elements (LINE-1 and B1 SINE) and of the endogenous retrovirus mysTR in the genomes of the three species. The results led us to infer that transposable elements have played an important role in the enormous chromosome variation found in Akodontini.

## 1. Introduction

Akodontini rodents comprise around 83 living species allocated in 15 genera [1]. Although they present a primarily Andean distribution, they are found throughout South America [1]. The genus *Akodon* is one of the most complex and specious within Sigmodontinae and is represented by 38 described species divided into five groups: *aerosus*, *boliviensis*, *cursor*, *dolores*, and *varius* [1].

Cytogenetic data have been very useful in species identification and in clarifying some systematic problems in *Akodon* [2]. Furthermore, species of this genus have highly variable karyotypes, with diploid numbers ranging from 2n = 9-10 in *Akodon* sp. n. to 2n = 44 in *A. mystax*, *A. paranaensis*, and *A. reigi* [1, 3]. Most of this karyotypic variation has been attributed to pericentric inversions and centric fusions, evidenced by comparative GTG- and CBG-banding, location of telomeres by in situ hybridization, and chromosome painting. The presence of supernumerary chromosomes and sex-chromosome heteromorphisms has also been reported [2, 4–9].

*Akodon cursor* (ACU) presents variation in diploid numbers (2n = 14 to 16) due to a complex rearrangement involving chromosomes 1 and 3, in which pericentric inversions followed by a centric fusion gave rise to a karyotype with 2n = 15 when in heterozygosis or 2n = 14 when in homozygosis [6, 8]. This species also presents variation in the fundamental numbers (FN = 18 to 26) due to pericentric inversions in pairs 2, 4, and 6 [10]. *Akodon montensis* (AMO) has a basic 2n = 24, but may show higher diploid numbers (2n = 25-26) due to the addition of B chromosomes [11, 12]. *Necromys lasiurus* (NLA) has 2n = 34, but some specimens showed 2n = 33 due to a heterozygous Robertsonian translocation between chromosomes 6 and 7 [7, 13, 14].

Chromosome painting with whole chromosome-specific probes from *Akodon* sp. n. (2n = 10), *A. cursor* (2n = 14, 15), *A. montensis* (2n = 24), and *A. paranaensis* (2n = 44) revealed that these species have undergone a recent process of rapid and extensive autosomal rearrangements revealed by the complete homology among their euchromatic portions and including complete conservation of the Y chromosome [9].

*Akodon* and *Necromys* have been recognized as closely related genera based on mitochondrial DNA sequences and comparative GTG-banding and are believed to have diverged around 3.55 million years ago (MYA) [7, 15, 16]. Interspecific chromosome homeology among *Akodon* species and *N. lasiurus* is considered high, but there is no information available on their heterochromatic components, which may have played a role in their genome differentiation.

Transposable elements (TEs) are one of the most abundant components of the heterochromatin and can play an important role in genomic diversity and evolutionary changes due to their high activity in transposition and recombination [17]. Many studies have demonstrated the presence of the retrotransposons LINE-1 (L1) and B1 SINE (B1) in mammals and rodents, respectively. However, some studies have shown an expansion of an endogenous retrovirus (mysTR) and inactivation of L1 and B1 in Sigmodontinae [18–22].

L1s evolved early during mammalian radiation and are present in marsupial and placental mammals [23]. They are considered important in X chromosome inactivation during female embryogenesis, and some species show preferential L1 accumulation on their X chromosomes. L1s have also been implicated in DNA repair, in gene expression regulation, and in self-mobilization, as well as in that of other sequences such as pseudogenes and SINEs [23–26]. L1s may also provide sites for ectopic recombination that lead to genome rearrangements, increasing the genetic diversity of a population [27]. L1s may be found in all chromosomes of a species, although most eutherian Y chromosomes do not exhibit these elements [24, 28–30]. They have been associated to AT-rich regions producing a GTG-banding-like pattern in some Euarchontoglires (human, murid rodents, and rabbits), but have not been found in the heterochromatin [24, 28–30]. On the other hand, L1s did not produce banding pattern in Afrotheria, Xenarthra, and Laurasiatheria [30].

B1s are short nonautonomous elements and, as many SINEs, may contribute to maintaining the stability and function of the host genome [31]. They are able to cause genome expansion through unequal crossover between copies and may also have roles in gene activity regulation, chromatin organization, and mutagenesis by retrotransposition within genes [32, 33]. SINEs are usually found in gene-rich GC regions and do not accumulate on the sex chromosomes [24, 32, 34].

Recently, Gualtieri et al. [35] demonstrated L1 and B1 copy number amplification and increased expression during murine mammary carcinoma progression, and the large number of TE copies was associated with a high chromosomal instability, favoring tumor progression.

The endogenous retrovirus mysTR was originally identified in the white-footed mouse *Peromyscus leucopus* [35]. These sequences are primarily located in AT-rich regions, accumulate mainly on the X and Y chromosomes, and appear to be absent from the satellite DNA-rich heterochromatin [28, 29, 35, 36]. It is known that endogenous retroviruses may represent a substantial source of genomic variation and promoters, may cause rearrangements by ectopic recombination, and may disrupt gene regulation [37].

In this work, we aimed to test the involvement of repetitive sequences in the karyotypic evolution of Akodontini. In order to do that, we performed comparative genomic analyses among *A. cursor*, *A. montensis*, and *Necromys lasiurus* based on GTG- and CBG-banding patterns, FISH with total genomic DNAs (GISH), and with telomeric sequences. We also examined the distribution of the transposable elements L1, B1, and mysTR in the chromosomes of the three species to assess their relationship to the karyotypic variation.

## 2. Materials and Methods

The specimens analyzed are listed in Table 1. They were collected in the state of Minas Gerais, Brazil, under the permits 12989-2, 14868-1, and 14868-2 from SISBIO-IBAMA conceded to Adriano Pereira Paglia and Edeltrudes MVC Câmara. The skulls and skins were deposited at the Museu de Ciências Naturais–Pontifícia Universidade Católica (PUC) (MCN-M) and in the mammalian collection of the Centro de Coleções Taxonômicas–Universidade Federal de Minas Gerais (UFMG), both in Belo Horizonte, Minas Gerais, Brazil. All the experiment design was derived from NPA's Master's dissertation [38]. Cytogenetic analyses were performed on chromosome preparations obtained directly from the bone marrow [39]. GTG- and CBG-banding patterns were performed according to [40, 41], respectively.

Genome comparisons among males of *Akodon cursor*, *A. montensis*, and *Necromys lasiurus* were performed by FISH with total genomic DNA extracted from the liver and labeled by Nick translation with digoxigenin-11-dUTP (DIG-Nick Translation Mix, Roche Applied Science), according to [42]. The schematic representation of the experiments performed is shown in Supplementary Table 1 available online at https://doi.org/10.1155/2017/5935380. In the control experiments, probes of each species were hybridized to the chromosomes of the same species, allowing to check the efficiency of the probes and of the experiment conditions. In order to test the suppression conditions (suppressor DNAs control), total labeled DNA and unlabeled genomic DNA of each species (proportion 1 : 100) were preannealed at 37°C for an hour and hybridized to the chromosomes of the same species. The hybridization mix with labeled genomic DNAs of each species and the mix probe:suppressor DNA were applied to the chromosome preparation of the other two species in order to check which genomic segments were common to both species and which were species-specific, respectively. The analyses were performed under a Zeiss Axioimager 2 epifluorescence microscope, and the images were captured with the AxioVision software (Zeiss).

A biotinylated telomeric sequence (TTAGGG)_4_ (Invitrogen) was synthesized and used as a probe for FISH. The hybridization mix, consisting of 1040 ng of probe in 50% formamide/2× SSC, was applied to the denatured chromosomes. Hybridization was carried out at 42°C overnight, immunodetection was performed with avidin-FITC (Roche Applied Science) and counterstaining with propidium iodide.

L1, B1, and the endogenous retrovirus mysTR were amplified by PCR from the genomic DNAs of the three species with the following primer sets: L1-F (5′AAGAATTCCGCAGGATACAAGATCAACTCA3′) and L1-R (5′AAGGATCCCAATTCGATTCCATTGGT3′) [20]; B1-F (5′GCCGGGCGTGGTGGCG3′) and B1-R (5′TTGGTTTTTCGAGACAGGGTTTCT3′) [21]; mysTR-F (5′ACGAATTGCTCGAGAGKIHTIITNGAYCANGG3′) and mysTR-R (5′ TGGATCGCTGCGGTARNADRTCRTCCATRTA3′) [22]. All PCR reactions consisted of an initial denaturation step of 94°C for 3 min and a final extension at 72°C for 10 min. Between these steps, 30 cycles were performed at 94°C for 60 s, 40°C for 60 s, and 72°C for 90 s (for L1 and mysTR) and 94°C for 60 s, 55°C for 60 s, and 72°C for 90 s (for B1). PCR products were purified with the Wizard SV Gel and PCR Clean-up System kit (Promega) and cloned into the pGEM-T Easy Vector kit (Promega). Recombinant plasmids were sequenced on the ABI3130 platform (Myleus Biotechnology). The sequences generated in this study have GenBank accession numbers KY701525 (L1), KY701526 (B1), and KY701527 (mysTR). Sequenced plasmids were labeled by nick-translation with digoxigenin-11-dUTP (DIG-Nick Translation Mix, Roche Applied Science) and used as probes for FISH. The hybridization mix consisted of 200 ng of digoxigenin-labeled probe, and the hybridizations were carried out at 42°C overnight. After posthybridization washes and immunodetection with antidigoxigen conjugated with rhodamine, the metaphases were counterstained with DAPI (0.8 ng/*μ*L) in antifade reagent (SlowFade, Invitrogen).

## 3. Results and Discussion

### 3.1. Interspecific Chromosome Rearrangements

A comparative analysis of the GTG-banded chromosomes of *Akodon cursor* (ACU, 2n = 14, FN = 19), *A. montensis* (AMO, 2n = 24, FN = 42), and *Necromys lasiurus* (NLA, 2n = 34, FN = 34) allowed us to establish a complete homeology among most chromosome arms of the three complements (Supplementary Figure 1; Table 2). The two ACU males had a heteromorphic pericentric inversion on pair 4, which was metacentric/acrocentric, explaining the odd FN. Most chromosomes arms showed complete correspondence among the three species. However, it was not possible to establish the correspondence of part of ACU 2 and of the entire AMO 6 to any NLA chromosomes (Supplementary Figure 1, Table 2). Our results agree with previous findings [4, 7–9].

Centric fusions explain the differentiation of some chromosomes: NLA 6 and 7 correspond to AMO 3 and to part of ACU 2; NLA 4 and 3 are homeologous to the short and long arms of ACU 4 and AMO 2, respectively; and NLA 8 and 12 correspond to ACU 6 and AMO 4. ACU 1 + 3 corresponds to AMO 1, 7, 8, and 9 and to NLA 2, 5, 9, 10, 11, 13, and 14, but pericentric inversions are also involved in the differentiation of these chromosomes. Robertsonian rearrangements followed by pericentric inversions were proposed as the primary mechanisms involved in the karyotypic evolution of these rodents [7].

Changes in chromosome morphology without apparent variation in GTG-banding patterns were observed between the metacentric AMO 5 and the acrocentric NLA 1 and also between the metacentric AMO 9 and the acrocentric NLA 9. These observations suggest that centromere repositioning and/or pericentric inversions may explain these chromosome differences. Furthermore, centromere repositioning and centric fusion are probably involved in the differentiation of ACU 5 from AMO 5 and 10 and NLA 15.

**Figure 1:**
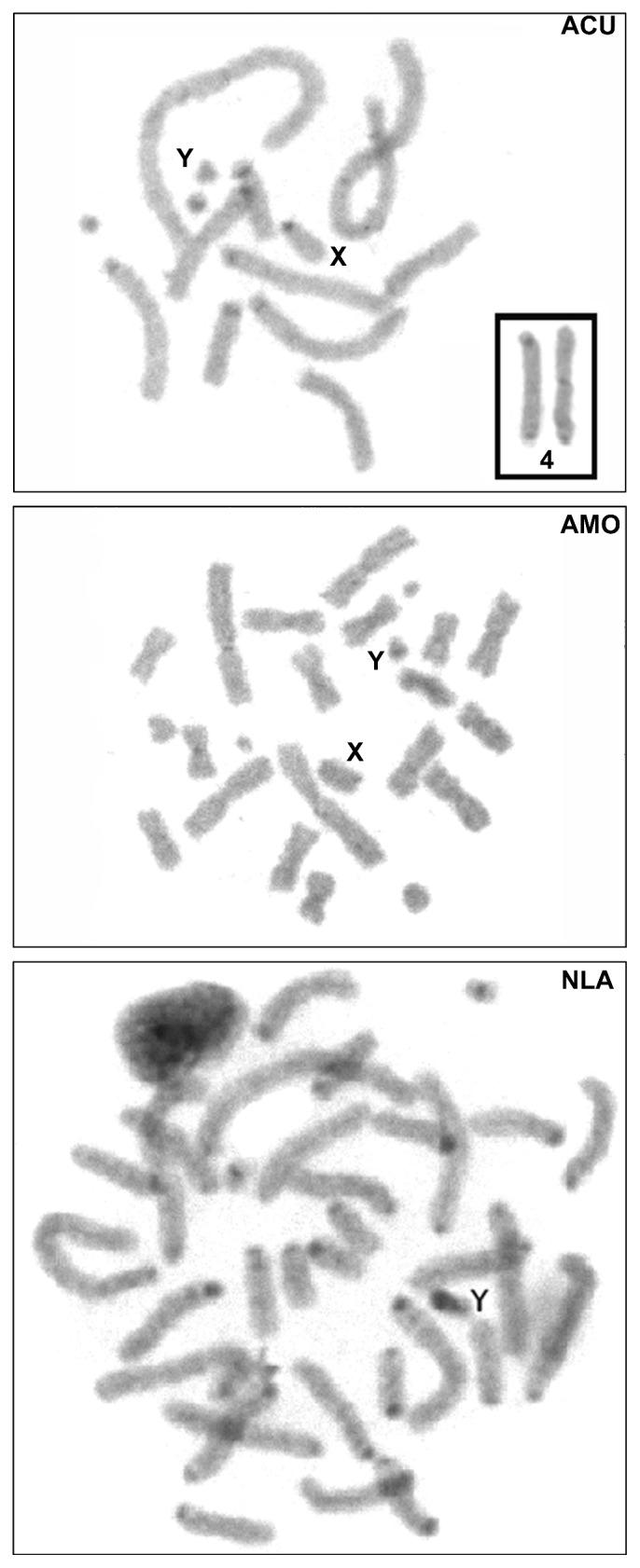
CBG-banded cells of *Akodon cursor* (ACU, 2n = 14), *A. montensis* (AMO, 2n = 24), and *Necromys lasiurus* (NLA, 2n = 34). ACU pair 4 is shown in the inset.

CBG-banding (Figure 1) in ACU revealed constitutive heterochromatin in the centromeric regions of all chromosomes, except in pair 5 and in the Y chromosome. Pair 4 also had heterochromatic telomeric regions. AMO had weak CBG-bands in the centromeric constitutive heterochromatin of all the autosomes and the X chromosome, while the Y chromosome was almost entirely heterochromatic (Figure 1). In NLA, centromeric CBG-bands were present in all autosomes and in the X chromosome. The Y chromosome was almost completely heterochromatic (Figure 1).

**Figure 2:**
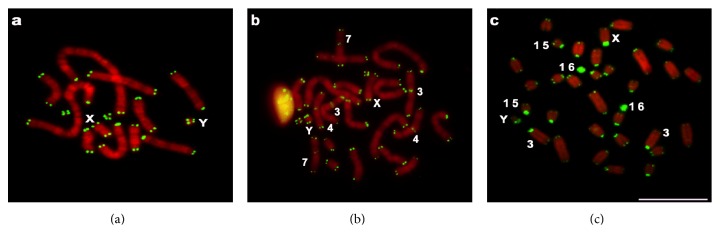
FISH with a telomeric probe in (a) *Akodon cursor* (2n = 14, FN = 19); (b) *A. montensis* (2n = 24, FN = 42); and (c) *Necromys lasiurus* (2n = 34, FN = 34). Bar = 10 *μ*m.

FISH with the telomeric probe yielded signals at both telomeres of each chromosome in the three species analyzed. No additional signals were found in ACU (Figure 2(a)). This result differs from those of Fagundes et al. [6, 8], in which interstitial telomeric sequences (ITSs) were found in the largest pairs of the karyotypes with 2n = 14 and 2n = 15. The presence of ITSs led the authors to suggest that pair 1 in the 2n = 14 karyotype originated after a pericentric inversion and a centric fusion occurred in an ancestral karyotype with 2n = 16.

The AMO karyotype had ITSs on the centromeric regions of pairs 3, 4, and 7 (Figure 2(b)). These sites correspond to fusions/fissions involved in the differentiation of the AMO and NLA complements (Figure 2(b), Table 2). AMO 3 corresponds to NLA 6 and 7, AMO 4 to NLA 8 and 12, and AMO 7 to NLA 14 and part of NLA 2. FISH with a telomeric probe on AMO chromosomes has been previously performed, and no ITSs were reported [7]. On the other hand, the presence of an IT on the metacentric NLA 6 + 7 of the karyotype with 2n = 33, which corresponds to AMO 3, was interpreted as resulting from a recent rearrangement [7].

NLA chromosomes displayed large telomeric signals on the centromeric regions of pairs 3 and 15 and on the sex chromosomes. In addition, pair 16 hybridized throughout its length (Figure 2(c)). Fagundes and Yonenaga-Yassuda [7] also found variation in the intensity of telomeric signals near the centromeres in NLA, mostly on the X chromosome. A similar pattern was also observed in *Akodon lindberghi*, which presented strong signals on the pericentromeric regions of the autosomes [43]. These results point to the presence of (T_2_AG_3_)_n_ sequences in the heterochromatin of NLA 3, 15, X, and Y and in the euchromatin of pair 16, as already suggested [7].

Although ACU 7 and AMO 11 seem homeologous to NLA 16 after GTG-banding (Supplementary Figure 1), an IT was present only in NLA 16. AMO 10 also differed from NLA 15, and the X chromosomes of both *Akodon* species differed from the NLA X due to the presence of telomeric sequences in their pericentromeric regions (Figure 2).

The origin of ITSs is still debated, but it is thought that they may represent remnants of ancestral chromosome rearrangements, such as inversions and centric or tandem fusions [44, 45]. In *Akodon*, ITSs located on pericentromeric regions were also found in chromosome 1 of *Akodon* sp. [3] and pairs 4 and 5 of *A. dolores* [46]. In all these cases, the authors suggested that the ITSs represented remnants of fusions.

Amplification events may lead to the formation of large ITSs, whereas deletions may result in their absence or reduction in size, preventing their visualization after FISH [47]. This kind of events are likely the reason of the variable results obtained by different authors ([6, 8, 9], this work) in the ACU and AMO chromosomes. ITSs have also been suggested to be associated with nontelomeric repetitive sequences [44, 45], which seems to be the case of NLA.

### 3.2. Levels of Euchromatin and Heterochromatin Differentiation

The degree of conservation among the genomes of ACU, AMO, and NLA was assessed through interspecific GISH using total genomic DNAs as probes. Control experiments are presented as Supplementary Figure 2. Hybridization of the labeled DNA of each species with its own chromosomes (probe controls) resulted in labeling throughout all the chromosomes, with brighter signals in the CBG-banded constitutive heterochromatin and telomeric regions. The suppressor DNA control experiments showed complete absence of hybridization signals on the propidium iodide counterstained metaphases (Supplementary Figure 2).

**Figure 3:**
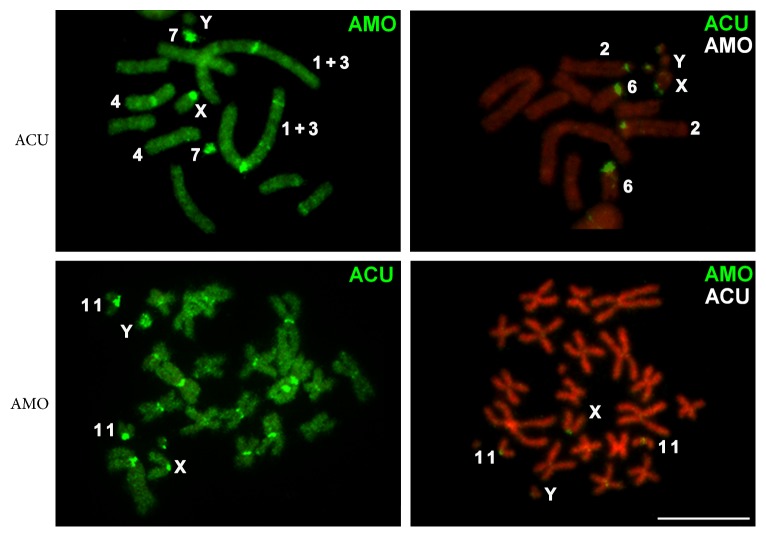
Interspecific GISH among *Akodon cursor* (ACU) and *A. montensis* (AMO). The initials on the left correspond to the species cells. The labeled DNA used is identified in green and the suppressor DNA is represented in white. All the cells were counterstained with propidium iodide. Bar = 10 *μ*m.

The interspecific hybridizations between ACU and AMO resulted in labeling of all euchromatic regions. The pericentromeric heterochromatin of ACU 1, 4, and X and of all AMO chromosomes, as well as the entire ACU 7, showed bright signals (Figure 3), revealing the presence of sequences shared by both species. In the interspecific experiments with suppression, the heterochromatin of ACU 2, 6, and X and of AMO 11 and X showed labeling, suggesting that they contain species-specific sequences. Therefore, the heterochromatic pericentromeric regions of AMO 11 and of the X chromosomes of both species contain both shared and species-specific sequences (Figure 3). Interestingly, in the interspecific experiments, the Y chromosomes of ACU and AMO hybridized throughout their extension, suggesting a very similar DNA composition in both species. Ventura et al. [9] obtained similar results using interspecific hybridizations with flow-sorted Y chromosomes of ACU, AMO, *Akodon* sp. (2n = 10), and *A. paranaensis*, which led them to conclude that this chromosome is conserved in *Akodon* species.

**Figure 4:**
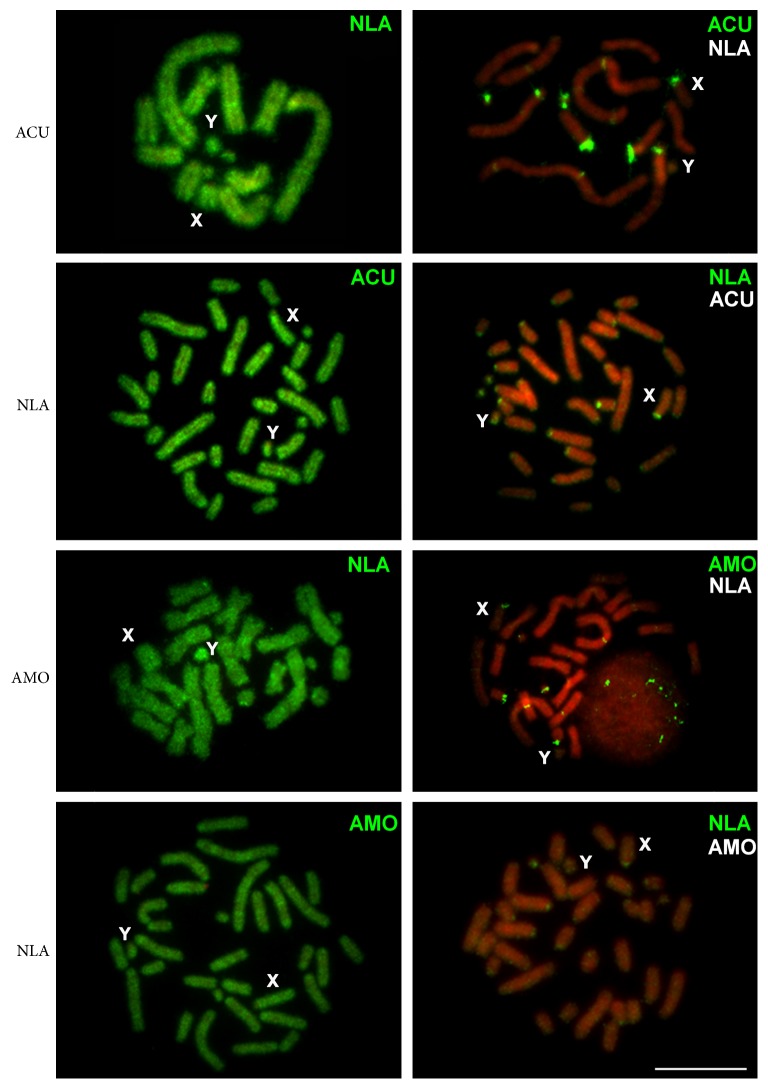
Interspecific GISH among *Akodon cursor* (ACU), *A. montensis* (AMO), and *Necromys lasiurus* (NLA). The initials on the left correspond to the species cells. The labeled DNA used is identified in green, and the suppressor DNA is represented in white. All the cells were counterstained with propidium iodide. Bar = 10 *μ*m.

Interspecific hybridizations with labeled DNAs of each *Akodon* species and NLA resulted in labeling of all euchromatic regions (Figure 4), suggesting a high conservation of these regions in the three species. On the other hand, the autosomal heterochromatic segments did not hybridize, pointing to their divergence. Hybridization experiments with suppression resulted in labeling of all the autosomal and X chromosomes constitutive heterochromatin of each species (Figure 4). Our experiments also evidenced that the Y chromosomes of both *Akodon* species and NLA seem to share great part of their content (Figure 4).

Using the flow-sorted *A. paranaensis* Y chromosome as probe, Ventura et al. [48] also demonstrated the conservation of Y euchromatic regions between this species and NLA. Together, these results contradict the commonly held notion that mammalian Y chromosomes are remarkably species-specific [49].

Comparative analyses of Y chromosomes are scarce in the literature, and the few examples of interspecific hybridizations using Y chromosome probes point to their specificity. For example, Acosta et al. [50] demonstrated a poor conservation of the Y chromosome among six arvicolid rodents. Among them, only the euchromatic Y chromosome region of *M. cabrerae* and *M. agrestis* shared similar sequences. The absence of conservation of the Y chromosome euchromatin could be a result of degenerative processes related to the evolution of this chromosome [51].

Although the genome contents seem conserved among the three analyzed species, the conservation of gene order along the chromosomes remains to be tested, for example, by mapping DNA markers through FISH.

### 3.3. Retrotransposons and Karyotypic Evolution

**Figure 5:**
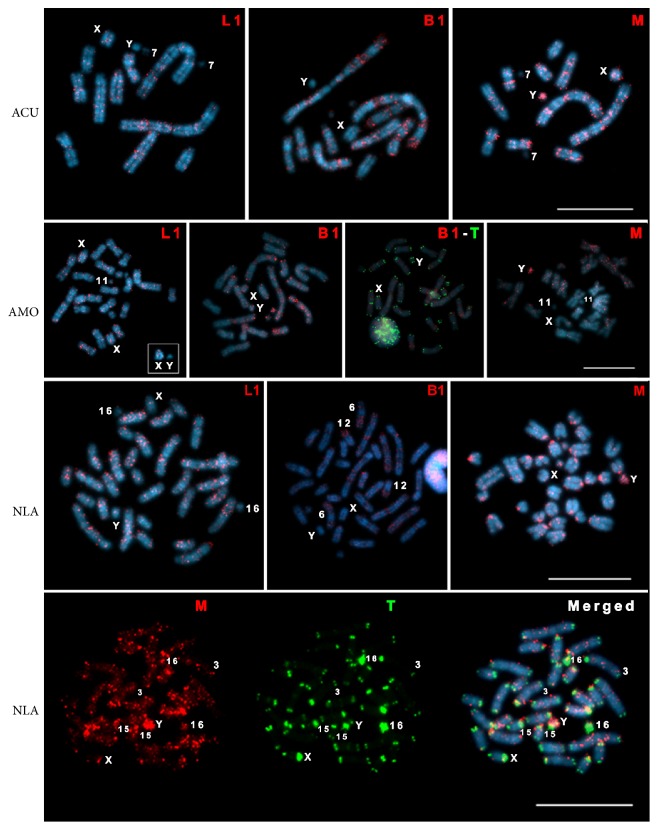
FISH with digoxigenin-labeled transposable elements and biotin-labeled telomeric sequences in cells of Akodontini. ACU: *Akodon cursor*; AMO: *A. montensis*; NLA: *Necromys lasiurus*; L1: LINE-1; B1: B1 SINE; M: mysTR, T: telomere sequence. The cells of AMO depicted are of a female and the sex chromosomes of a male are shown in the inset. Bar = 10 *μ*m.

PCR from genomic DNA of ACU, AMO, and NLA with primers specific for L1 and B1 resulted in amplicons of the expected sizes with approximately 500 bp and 150 bp, respectively. The PCR with primers specific for mysTR did not yield products for AMO and NLA, whereas a smear was obtained with ACU genomic DNA. For these reasons, we performed the same PCR using the genomic DNA of another species, *Akodon* sp. (2n = 44, FN = 46), and obtained amplicons of the expected size (~800 bp). After cloning and sequencing, we ended up with three clones representing the three retrotransposon sequences. These clones were labeled with digoxigenin and hybridized to the chromosomes of each species (Figure 5).

L1 sequences showed a dispersed distribution, but preferentially located to DAPI bright bands, which correspond to the AT-rich regions, in ACU, AMO, and NLA chromosomes. No hybridization signals were found in the constitutive heterochromatin and on the corresponding autosomes ACU 7, AMO 11, and NLA 16. The lack of L1 signals in the constitutive heterochromatin of ACU, AMO, and NLA resembles the results obtained in *Mus musculus* and *Peromyscus maniculatus* [24, 28, 29], suggesting that these TEs are not involved in the heterochromatin formation and maintenance in these species.

The Y chromosomes were devoid of hybridization signals and the X chromosomes presented few signals in the three species (Figure 5). These results differ from those obtained in the L1-active species *M. musculus*, *P. maniculatus*, and four *Taterillus* species in which a nonrandom GTG-banding-like L1 distribution was reported [24, 28, 29, 52]. The X and Y chromosomes of these species, differently from ours, were labeled by L1 throughout their lengths.

L1 accumulation on the X chromosome of eutherian mammals has been associated with chromosome inactivation during female embryogenesis [25, 30]. However, in Sigmodontinae, these sequences seem to have lost transposition activity around 8.8 MYA [18, 20–22], which may explain the few signals observed on the X chromosomes that we analyzed. It has been suggested that L1 interacts with XIST to silence genes on the inactive X [53], being thus involved in X inactivation through a mechanism different from the way stations proposed by Lyon [25]. Cantrell et al. [54] studied the relationship between L1 activity and X inactivation in the Sigmodontinae *Oryzomys palustris* and found that X-inactivation was normal even in the absence of L1 activity. This may also be the case in *Akodon*, as we did not find L1 accumulation in the X chromosomes of the analyzed species.

**Figure 6:**
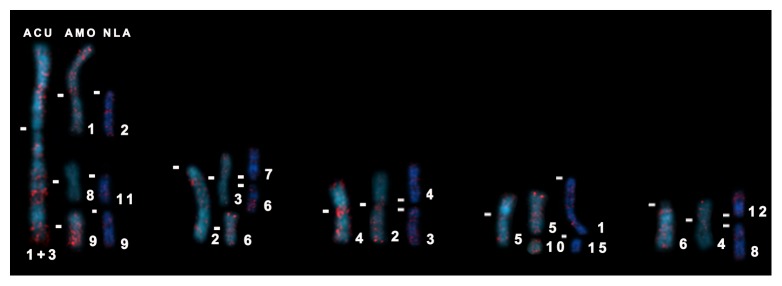
Correspondence between the B1 SINE-hybridized chromosomes of *Akodon cursor* (ACU), on the left, *A. montensis* (AMO), in the middle, and *Necromys lasiurus* (NLA), on the right. Chromosome correspondences were based on Supplementary Figure 1. (-) centromere position.

B1 elements preferentially hybridized to the GC-rich dull DAPI bands of the karyotypes of the three Akodontini species (Figure 5). B1 did not colocalize with L1 and was not preferentially accumulated on the sex chromosomes, as also reported for *M. musculus* [24]. Because B1 seemed to hybridize to telomeres in AMO (Figure 5), we performed double FISH with B1 and telomeric probes. This experiment revealed that B1 did not colocalize neither with the telomeres nor with the ITSs of AMO 3, 4, and 7 (Figure 5). Interestingly, B1 presented a nonrandom distribution with conserved patterns in some chromosomes of both *Akodon* species, but not in the corresponding NLA chromosomes (Figure 6). For example, the long arm of AMO 9 presented great accumulation of B1, similarly to the corresponding ACU 1 + 3 region, but B1 accumulation was absent from the corresponding region of NLA 9 (Figure 6). B1 also produced signals in the pericentromeric region of AMO 1 and on its corresponding segment on ACU 1 + 3, but not on the homeologous NLA 2. The corresponding chromosomes ACU 2 and AMO 3 and 6 presented a dispersed distribution of B1. On the other hand, NLA 6 presented a great accumulation of B1 that was not observed in its *Akodon* counterparts. The same pattern could be observed between ACU 6, AMO 4, NLA 8, and 12. ACU 4 and AMO 2 showed B1 signals at their pericentromeric regions, which were not seen on the corresponding NLA 3 and 4 segments. Furthermore, ACU 5 had signals at its pericentromeric region, as did its counterpart AMO 5. AMO 10 presented a dispersed B1 distribution, not observed in the corresponding NLA 15 (Figure 6).

All the chromosome regions pointed out above have been suggested as sites of fusions/fissions and pericentric inversions during the karyotypic evolution of these species. The accumulation of B1 in these regions allows us to hypothesize a relationship between these repetitive sequences and the occurrence of rearrangements. Indeed, TEs have been previously associated with chromosome rearrangements and with the induction of insertions and deletions [55, 56]. However, it is still an open question whether B1 accumulation prompted the rearrangements or if it occurred after they took place. Further analyses of B1 sequences in Akodontini may shed light on their involvement in the high degree of karyotypic change observed in these rodents.

MysTR sequences were located in bright DAPI bands of ACU, AMO, and NLA, and the Y chromosomes of these species showed preferential accumulation of this element (Figure 5). In both *Akodon* species, mysTR sequences did not hybridize to the heterochromatin and to chromosomes ACU 7 and AMO 11 (Figure 5). The absence of mysTR elements was also observed in autosomal CBG-banded regions of some *Peromyscus* species [28, 29, 36]. On the other hand, labeling occurred in the heterochromatic regions of most NLA chromosomes (Figure 5). In this species, double-FISH with telomeric and mysTR sequences as probes revealed a few colocalizations in some autosomes and in the transition between the euchromatic and heterochromatic portions of the Y chromosome (Figure 5), although there is no sequence similarity between mysTR and the telomeric (TTAGGG)_n_. FISH on metaphase chromosomes yield distinct signals for sequences separated by at least 1 Mb [57]. Therefore, the seemingly colocalization of mysTR and (TTAGGG)_n_ probably results from a technical constraint. Our data represent the first demonstration of mysTR sequences in *Necromys.*

According to Cantrell et al. [19], mysTR showed a dispersed distribution throughout all chromosomes of *Oryzomys palustris*, but the authors did not mention their location on the sex chromosomes. A preferential accumulation of mysTR on the Y chromosome, similar to that observed in our specimens, has also been reported in *Peromyscus* species, which, differently from the species analyzed herein, also accumulated mysTR on the X [28, 29, 36]. Chromosome painting with Y-specific probes in ten Akodontini species revealed interspecific homologies of some segments [9, 48]. We obtained similar results with the GISH experiments (Figures 3 and 4). Additionally, we observed strong hybridization signals on the Y chromosomes of *Akodon* and *Necromys* with the mysTR probe. These observations suggest that the Y chromosome portion shared by Akodontini species may actually represent mysTR sequences. The hybridization of mysTR in additional Akodontini should help to test this hypothesis.

Retroviruses depend on the host cell replication to integrate into the genome [37]. Thus, a larger number of cell divisions in the male germ line could explain the preferential accumulation of the endogenous retrovirus mysTR on the Y and not on the autosomes and X chromosomes of Akodontini, as suggested for human Y-chromosome retroviruses [58]. In addition, endogenous retrovirus accumulation could result from the lack of Y chromosome recombination.

## 4. Conclusions

Our results showed great conservation of euchromatic regions among the karyotypes of *Akodon cursor*, *A. montensis*, and *Necromys lasiurus*. Besides Robertsonian rearrangements and pericentric inversions, we also propose that centromere repositioning may be involved in the karyotype differentiation. The analyses of three TEs yielded some important results: L1 is not accumulated in the X chromosome suggesting that it is not involved in this chromosome inactivation in Akodontini. MysTR is preferentially located on the Y chromosome of the three studied species, which may explain the Y chromosome conservation observed after interspecific chromosome painting in Akodontini. B1 was mainly found at putative interspecific rearrangement sites, suggesting its possible relationship with the great chromosomal variability of Akodontini and points to the need of further studies of B1 in this clade.

## Supplementary Material

The information of supplementary materials are as follows: Supplementary Table 1 - Schematic representation of genomic comparisons through fluorescent in situ hybridization with total genomic DNA (GISH). Supplementary Fig. 1 Correspondence between the GTG-banded chromosomes of Akodontini. Akodon cursor (ACU, 2n=14), on the left, A. montensis (AMO, 2n=24), in the middle, and Necromys lasiurus (NLA, 2n=34), on the right. Supplementary Fig. 2 Control GISH experiments of: (a) Akodon cursor (2n=14, FN=19), (b) A. montensis (2n=24, FN=42), and (c) Necromys lasiurus (2n=34, FN=34). Control suppression experiments of: (d) A. cursor, (e) A. montensis, and (f) N. lasiurus. Chromosomes were counterstained with propidium iodide. Bar = 10 µm.

## Figures and Tables

**Table 1:** Specimens analyzed.

Species	2n	FN	Collection sites	Deposit numbers (sex)
*Akodon cursor*	14	19	Conceição do Mato Dentro/MG (19°02′13″S 43°25′30″W)	MCN-M 2249 (M)
			Rio Pomba/MG (21°16′ 30″ S 43° 10′ 44″ W)	UFMG 6025 (M)
*Akodon montensis*	24	42	Morada Nova de Minas/MG (18°36′14″S 45°21′25″W)	MCN-M 2277 (M)
			Catas Altas (20°04′30″S 43°24′28″W)	MCN-M1586 (F)
*Akodon* sp.	44	46	Santana do Riacho/MG (19°10′08″S 43°42′50″W)	MCN-M 986 (M)
*Necromys lasiurus*	34	34	Augusto de Lima/MG (18°06′32″S 44°16′01″W)	UFMG 3836 (M)

2n: diploid number; FN: fundamental number; M: male; F: female; MCN-M: Museu de Ciências Naturais–Pontifícia Universidade Católica (PUC), Minas Gerais; UFMG: Centro de Coleções Taxonômicas–Universidade Federal de Minas Gerais (UFMG), Minas Gerais.

**Table 2:** Correspondence of GTG-banded chromosomes of *Akodon cursor* (ACU; 2n = 14, FN = 19), *A. montensis* (AMO; 2n = 24, FN = 42), and *Necromys lasiurus* (NLA; 2n = 34, FN = 34).

NLA	5	14	7	6	?	4	3	1	15	12	8	16	X
	10	13											
	2	11											
		9											

AMO	1	7	3p	3q	6	2p	2q	5	10	4p	4q	11	X
		8											
		9											
ACU	1p	1q	2			4^∗^p	4^∗^q	5		6		7	X

∗The metacentric chromosome was used for comparison; p = short arm; q = long arm.
