# Supplementary material for: Comparative Genomic In Situ Hybridization and the Possible Role of Retroelements in the Karyotypic Evolution of Three Akodontini Species
Source: Int J Genomics. 2017 Aug 15;2017:5935380. doi: 10.1155/2017/5935380 (PMC5576401; doi:10.1155/2017/5935380)
Supplement: Supplementary file 2 [file 5935380.f2.pdf]

Supplementary Material

Supplementary Table 1 - Schematic representation of genomic comparisons through fluorescent *in situ* hybridization with total genomic DNA (GISH).

Experiments*	Labeled DNA	Suppressor DNA	Chromosomes
I	Species A	-	Species A
II	Species B	-	Species B
III	Species A	Species A	Species A
IV	Species B	Species B	Species B
V	Species A	-	Species B
VI	Species B	-	Species A
VII	Species A	Species B	Species A
VIII	Species B	Species A	Species B

*Experiments:

I and II: probe control.

III and IV: suppressor DNA control.

V and VI: segments common to both species.

VII and VIII: species-specific segments.

In the control experiments, 250 ng of labeled total genomic DNAs in 50% formamide/2xSSC were hybridized to the chromosomes of the same species, allowing to check the efficiency of the probes and of the experiment conditions. In the three species these experiments resulted in labeling throughout all the chromosomes, with brighter signals in the CBG-banded constitutive heterochromatin and telomeric regions (Supplementary Fig. 2). In order to test the suppression conditions, total labeled DNA and unlabeled genomic DNA of each species (proportion 1:100) were preannealed at

37ºC for an hour and hybridized to the chromosomes of the same species. These experiments resulted in the complete absence of hybridization (Supplementary Fig. 2). In the suppression experiments, the mix probe:suppressor DNA were applied to chromosome preparations of each species. Hybridizations were carried out at 37ºC for three days and post-hybridization washes consisted of one 2xSSC bath at 42ºC. Immunodetection was performed with antidigoxigenin conjugated with FITC (Roche Applied Science). The chromosome preparations were counterstained with propidium iodide (0,6ng/µL) and mounted with DAPI (0,8ng/µL) in antifade reagent (Slowfade, Invitrogen).

Supplementary Figure 1

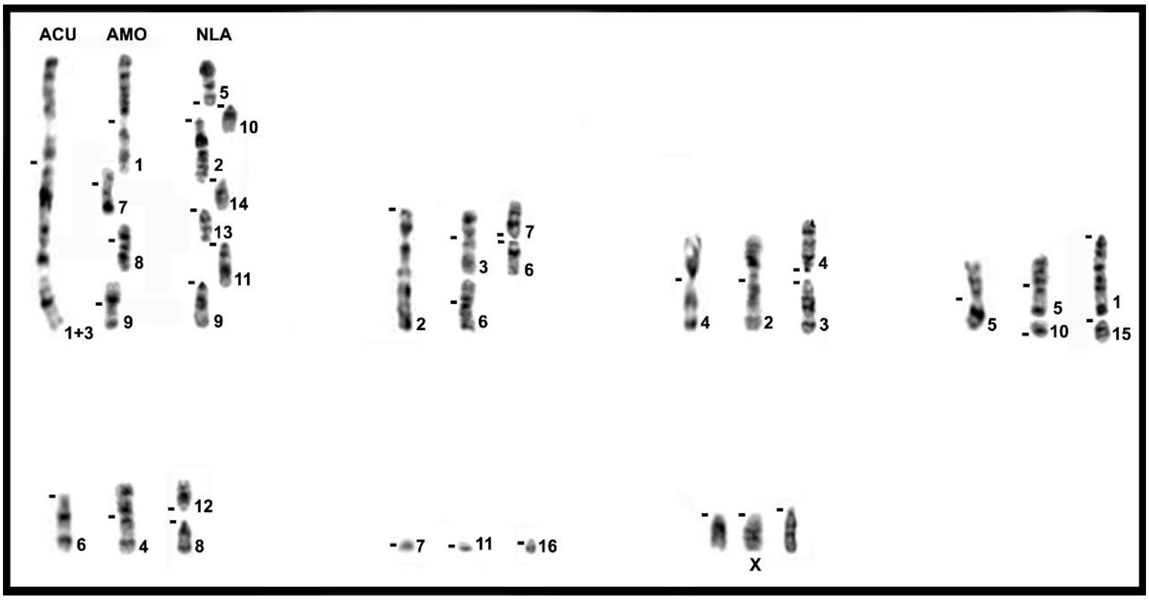

Supplementary Fig. 1 Correspondence between the GTG-banded chromosomes of Akodontini. *Akodon cursor* (ACU, 2n=14), on the left, *A. montensis* (AMO, 2n=24), in the middle, and *Necromys lasiurus* (NLA, 2n=34), on the right.

Supplementary Figure 2

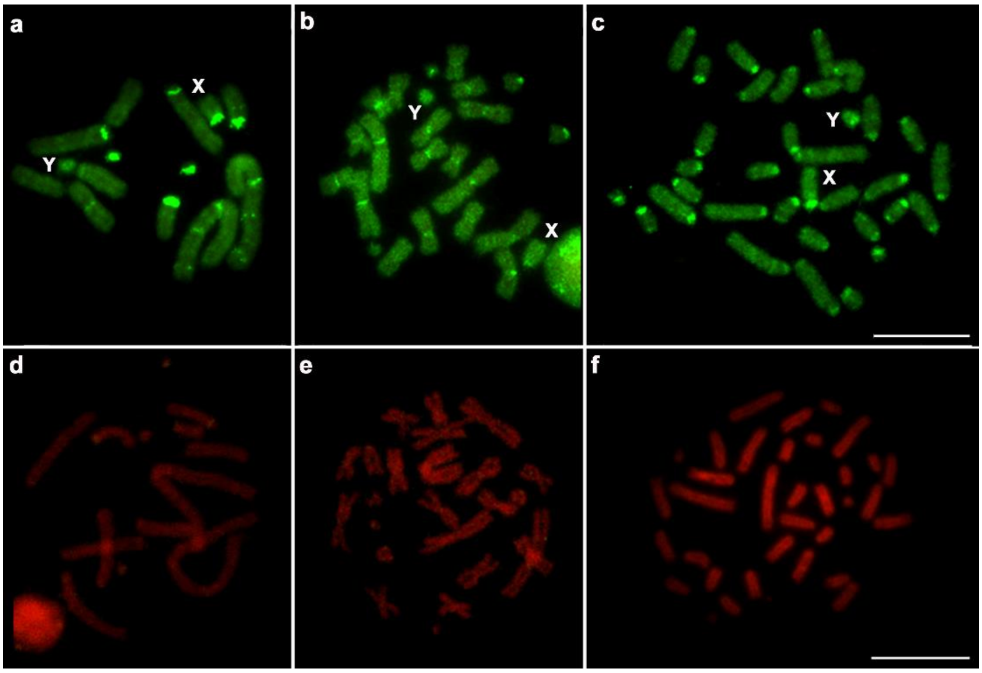

Supplementary Fig. 2 Control GISH experiments of: (a) *Akodon cursor* (2n=14, FN=19), (b) *A. montensis* (2n=24, FN=42), and (c) *Necromys lasiurus* (2n=34, FN=34). Control suppression experiments of: (d) *A. cursor*, (e) *A. montensis*, and (f) *N. lasiurus*. Chromosomes were counterstained with propidium iodide. Bar = 10 µm.
